# Transabdominal preperitoneal laparoscopic approach for incarcerated inguinal hernia repair: A report of 73 cases

**DOI:** 10.1097/MD.0000000000005686

**Published:** 2016-12-30

**Authors:** Shuo Yang, Guangyong Zhang, Cuihong Jin, Jinxin Cao, Yilin Zhu, Yingmo Shen, Minggang Wang

**Affiliations:** a Department of Hernia and Abdominal Wall Surgery, Beijing Chao-Yang Hospital, Capital Medical University, Beijing; b Department of Hernia and Abdominal Wall Surgery, Qilu Hospital of Shandong University, Jinan, Shandong, China.

**Keywords:** hernia, herniorrhaphy, inguinal, laparoscopy, TAPP

## Abstract

To investigate the efficacy, key technical points, and complication management of the transabdominal preperitoneal (TAPP) approach for incarcerated inguinal hernia repair. Seventy-three patients with incarcerated inguinal hernias underwent TAPP surgery in our department between Jan 2010 and Dec 2015. A retrospective review was performed by analyzing the perioperative data from these patients. The operation was successfully completed in all 73 patients. Operation time was 54.0 ± 18.8 minutes (range, 35–100 minutes). Length of stay was 3.9 ± 1.1 days (range, 3–9 days). There was 1 case of incisional infection, 32 cases of seroma, and 3 cases of postoperative pain during follow-up. All patients recovered after the appropriate treatment. No recurrence or fistula was observed. The TAPP approach represents a safe and effective technique for incarcerated inguinal hernia repair because of its potential in assessment of hernia content and decreasing incisional infection rate. However, it requires experienced surgeons to ensure safety with special attention paid to the key technical points as well as complication management.

## 1 Introduction

Incarcerated inguinal hernia is a life-threatening clinical emergency.^[1]^ Based on previous reports, about 10% of inguinal hernias and 20% of femoral hernias can become incarcerated, with a relatively higher complication rate than that of nonincarcerated hernias.^[2]^ Therefore, regardless of the site and size, emergency surgery is usually the first choice for an incarcerated hernia. However, there remain many uncertainties, and surgical options are still controversial. Since the transabdominal preperitoneal (TAPP) approach has advantages in assessing hernia content and decreasing incision infection rate, some researchers have started to use the TAPP approach for incarcerated inguinal hernia repair. However, therapeutic outcomes have not been validated.^[3]^ In the present report, we conducted a retrospective analysis to evaluate the efficacy of the TAPP approach for incarcerated inguinal hernia repair, and further discuss key technical points and complication management.

## 2 Methods

From Jan 2010 to Dec 2015, data from 73 patients diagnosed with incarcerated inguinal hernia (Table 1) undergoing a repair using the TAPP approach in the Department of Hernia and Abdominal Wall Surgery, Beijing Chao-Yang Hospital were retrospectively analyzed. The protocol was approved by the Ethics Committee of Beijing Chao-Yang Hospital, Capital Medical University.

**Table 1:**
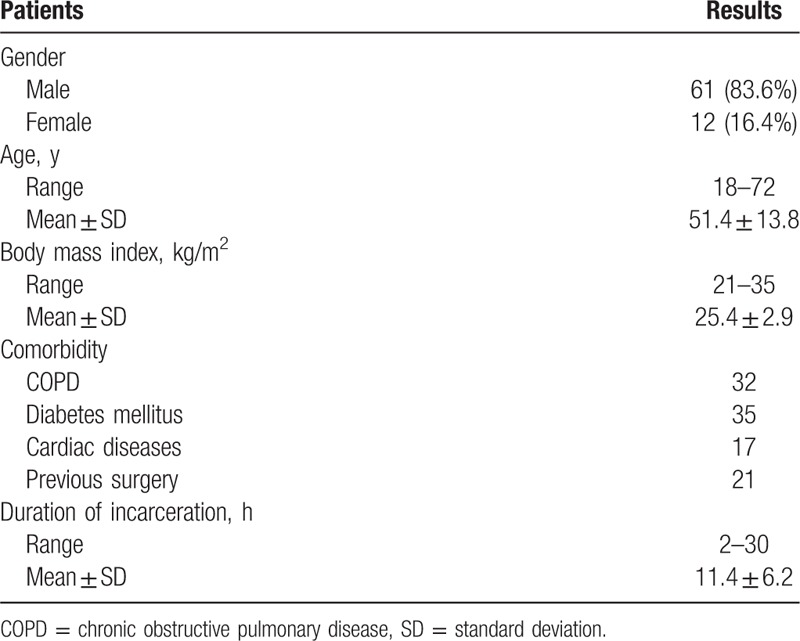
Baseline characteristics of individuals.

Case inclusion criteria were as follows: adult patients over 18 years old, having an incarcerated hernia, having no severe infectious disease diagnosed before surgery, and normal cardiopulmonary function and American Society of Anesthesiologists (ASA) I or ASA II.

Case exclusion criteria were as follows: the presence of a recurrent hernia or previous treatment with preperitoneal tissue adhesives.

All surgeries were performed under complete general anesthesia by a single surgeon. The clinical conditions and surgical risks were preoperatively evaluated by the same anesthesiologist. Prophylactic antibiotics were administered in all cases, and a postoperative antibiotic treatment was performed only for strangulated hernias. The antibiotics used were cefoxitin or alternatively levofloxacin, if the patients were allergic to cefoxitin. The TAPP approach was applied in all cases. A pneumoperitoneum was built via a Veress needle, and CO_2_ pressure was set at 14 mm Hg. A 10-mm optical trocar was inserted directly from the skin (just above the umbilicus) into the abdomen without dermal incision, and 2 5-mm operating trocars were inserted at each midclavicular line (1 or 2 cm under the umbilicus). Then, an abdominal exploration was performed to identify the superficial anatomical landmarks (e.g., the urachus, umbilical folds, epigastric vessels, spermatic vessels, and vas deferens or round ligament of the uterus) as well as the site and type of hernia (Table 2). The contents of the hernia sac were released or resected, if necessary. The remaining steps were performed as per standard TAPP approach.^[4]^ The meshes used were mainly polypropylene (Polypropylene, Bard company, GA) and soft (Monofilament Polypropylene, Bard company) types. Biomaterial patches (Acellular matrix, Beijing Qingyuanweiye Bio, Beijing, China) were used for 2 young patients. All meshes were fixed using medical glue (n-butyl-cyanoacrylate, Compont medical devices, Changzhou, Jiangsu, China). The peritoneum was sutured continuously using 3-0 Prolene (Ethicon, Orange, CA). For strangulated hernia (confirmed by visual observation; presence of abdominal pain; and elevated white blood cell count, temperature, and pulse), open intestinal resection was performed and a soft mesh was used with a latex drainage catheter placed in the pelvic cavity at the end of surgery.

**Table 2:**
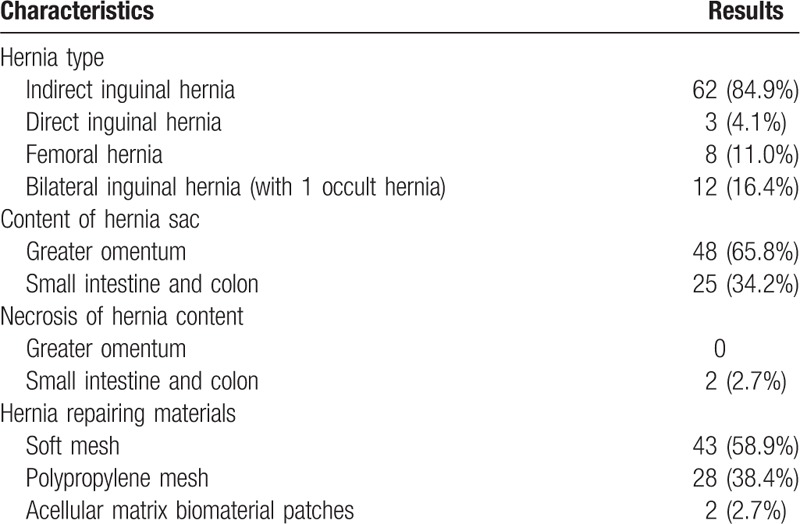
Characteristics of hernias and intraoperative findings.

Data are expressed as mean ± standard deviation.

## 3 Results

All surgeries were successful. The operation time was 54.0 ± 18.8 minutes (range, 35–100 minutes). The length of hospital stay was 3.9 ± 1.1 days (range, 3–9 days). The follow-up duration was 18.2 ± 13.1 months (range, 3–60 months) after the operation with a 100% follow-up rate.

During surgery, of 73 patients, 19 experienced an automatic hernia content reduction after initiation of anesthesia with the usage of muscle relaxants. Forceps-assisted pulling was needed in 45 patients, and hernia sac incision was performed in 9 patients. Two patients with strangulated hernia underwent partial small bowel resection due to necrosis; 1 of these patients had wound fat liquefaction, and the wound eventually healed after repeated dressing changes. Postoperative hematoma or seroma within the groin area was observed in 32 patients subsequent to indirect hernia repair. The seromas were absorbed without intervention within 2 weeks in 20 patients. Twelve patients showed swelling of the scrotum with a hydrocele over 30 mL (confirmed by ultrasound) at 2 weeks after surgery and were treated by puncturing the hydrocele. Three patients experienced postoperative pain (visual analog scale >3), which was relieved within 2 months after using low-dose painkillers (ibuprofen or aspirin) or undergoing physiotherapy. No recurrence or bowel fistula was observed (Table 3).

**Table 3:**
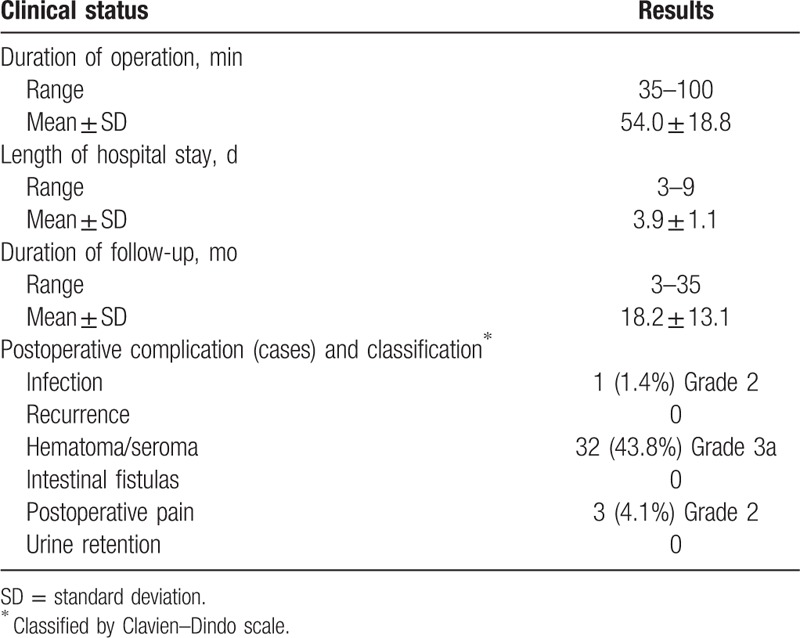
Clinical status.

## 4 Discussion

The TAPP approach is a laparoscopic technique with superior achievement of fast postoperative recovery compared to open surgery and is utilized in simultaneous bilateral hernia treatment as well as for assessing hernia content. However, conventional wisdom may suggest that the TAPP approach not be applicable to incarcerated hernia or strangulated hernia, because of its long learning curve and demanding skill requirements.

Currently, with a growing need for incessant improvement of surgical skills, we have started to use the TAPP approach for incarcerated hernia repair, because of its superiority in assessing gut viability. In open surgery, the incarcerated hernia content might return to the abdominal cavity due to the usage of narcotic drugs and muscle relaxants, and, accordingly, the incarcerated content always causes a dilemma for surgeons: should exploratory laparotomy be performed or not? Exploratory laparotomy searching for the incarcerated content might increase the risk of infection and extensive surgical trauma; however, if it is not performed, the potentially necrotic content might lead to bowel perforation, abdominal infection, and even deadly sepsis. Using the TAPP approach avoids this conundrum.^[5]^ It can be successfully performed in most cases of inguinal hernia. However, for incarcerated hernia, there are some differences because of its particular pathophysiology. Based on the experience of our department, we can provide some guidance on applying this surgical technique.

### 4.1 Key technical points in the TAPP approach for incarcerated inguinal hernia repair

#### 4.1.1 Hernia content reduction

**Figure 1:**
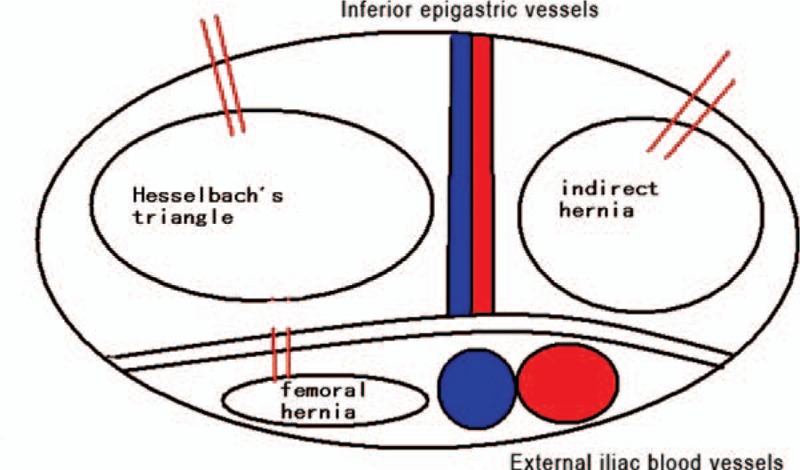
Demonstration of hernia sac opening procedure (double red lines indicate the direction for opening the hernia sac).

**Figure 2:**
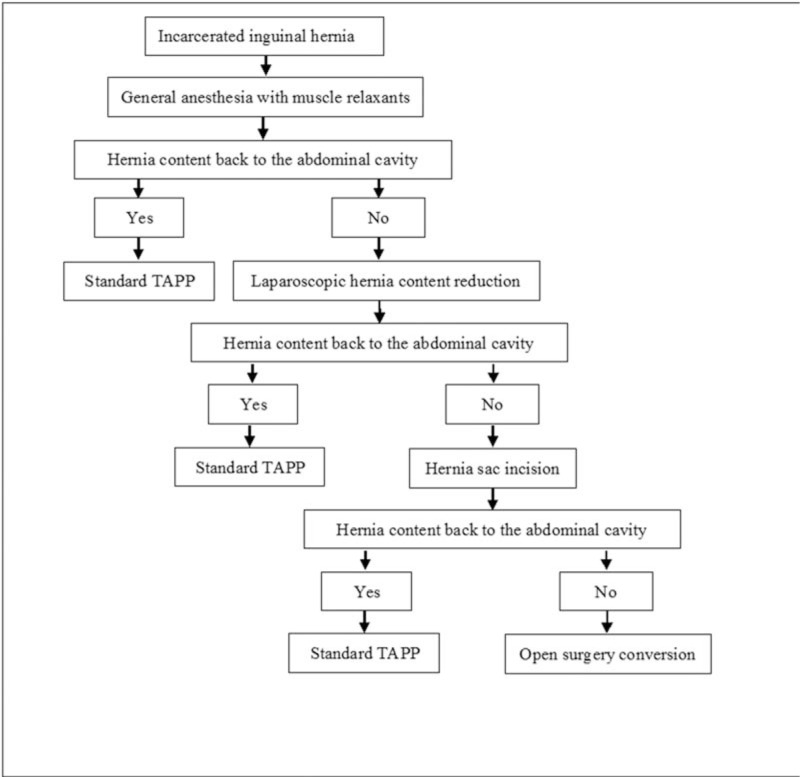
Schematic flow of hernia content reduction.

During the TAPP procedure, most of the incarcerated content might have returned to the abdominal cavity automatically due to the use of narcotic drugs, muscle relaxants, or the force of gravity.^[6]^ For those requiring intraoperative intervention, atraumatic forceps are strongly recommended to grasp the nonedematous mesenterium and pull back the incarcerated content, avoiding any bleeding or damage to the bowel. In case the incarcerated content cannot be reduced by simple pulling, the hernia sac should be instantly opened. For direct hernia, it is better to open the conjoined tendon or the outer rim of abdominal rectus muscle; for indirect hernia, we suggest dissecting from the upper lateral, which is safe and away from the inferior epigastric vessels as well as the triangle of doom; for femoral hernia, it is easier to open the inguinal ligament in the upper middle (Fig. 1). It should be noted that in some cases with long duration of hernia or previous usage of abdominal wall adhesives, there might be severe surrounding adhesions that cannot be dissected by simply opening the hernia sac. In this scenario, open surgery should be considered. After the hernia content returned into the abdominal cavity, the viability of the bowel should always be confirmed (Fig. 2).

#### 4.1.2 Preperitoneal and hernia sac dissection

The TAPP approach procedure recommends entering the peritoneum 1 to 2 cm above the inner ring. Nevertheless, in incarcerated cases, it is required to enter the peritoneum away from the inner ring, as the edematous tissues could lead to tardive rupture.^[7]^ For indirect hernia, we recommend resecting the sac 1 to 2 cm away from the inner ring, which may shorten the operative time.

### 4.2 Complication management in the TAPP approach for incarcerated inguinal hernia repair

#### 4.2.1 Infection

A mesh implantation for incarcerated hernia was previously considered as a contraindication, as the prosthesis may cause severe infection. In recent years, with further understanding of the anatomy of the groin area, improvement in surgical techniques, and better tissue compatibility of the hernia-repairing materials,^[8]^ an impressive body of evidence indicates that a tension-free herniorrhaphy with a full-size light mesh implantation is safe, based on an acceptable rate of infection and lower rate of recurrence.^[9]^ In the present report, of all 73 cases, incisional liquefaction only occurred in 1 strangulated case after converting to open surgery, and only 1 infection case was observed. These outcomes favor the feasibility and safety of the TAPP approach with mesh implantation for incarcerated inguinal hernia repair.

#### 4.2.2 Hematoma or seroma

Hematoma or seroma generally refers to a mass around the groin area, which may or may not extend to the scrotum, diagnosed by ultrasound, and fully absorbable in most cases.^[10]^ The distal sac of the indirect hernia was challenging to dissect completely and might leave small cavities, which could explain the high rate of hematoma or seroma after indirect hernia repair in the present report. It should be noted that careful observation of dynamic changes within the groin area and proper antibiotic treatment in some cases are necessary.

#### 4.2.3 Postoperative pain

Postoperative pain results from one or both of the following: nerve damage, contracture, and cicatrization of the implanted mesh.^[11]^ Regarding the former, using medical glue or a self-gripping mesh might provide better outcomes; for the latter, using a light-weight mesh might assist in reducing postoperative pain. Furthermore, tissue damage or postoperative edema could also result in postoperative pain.^[9]^ Therefore, specific attention should be paid to tissue dissection, particularly when resecting ligaments or dissecting a hernia sac.

It should be noted that the present study is a case series, based on experiences from a single institution. Further randomized controlled trials investigating TAPP approach for incarcerated inguinal hernia repair are still warranted.

## 5 Conclusion

The TAPP approach represents a safe and effective technique for incarcerated inguinal hernia repair because of its potential advantages in assessing hernia content and decreasing incisional infection rate. However, it requires experienced surgeons to ensure safety with special attention paid to the key technical points as well as complication management.
